# Ten-Year Risk for Developing Cardiovascular Disease Among Older Adults and Elderly in India: A Secondary Analysis of Wave-1 of Longitudinal Aging Study in India

**DOI:** 10.7759/cureus.46772

**Published:** 2023-10-10

**Authors:** Vignesh Loganathan, Muthathal Subramanian, Sitanshu Sekhar Kar

**Affiliations:** 1 Department of Preventive and Social Medicine, Jawaharlal Institute of Postgraduate Medical Education and Research, Puducherry, IND; 2 Department of Community and Family Medicine, All India Institute of Medical Sciences, Raipur, IND

**Keywords:** india, secondary analysis, lasi, risk prediction, cardiovascular diseases

## Abstract

Background

Cardiovascular disease (CVD) risk stratification is recommended by the World Health Organization (WHO) for effective CVD management in primary healthcare settings. Using the 2019 updated WHO CVD risk charts, we estimated the 10-year risk for developing fatal and non-fatal CVD among participants of the Longitudinal Aging Study in India (LASI).

Methods

We conducted secondary data analysis using the Wave-1 dataset of LASI. Analysis was performed in Stata software (version 14.1; StataCorp LLC, College Station, Texas) after applying sample weights. Ten-year CVD risk was estimated using a non-laboratory-based CVD risk chart. Logistic regression analysis was performed to determine the association between socio-demographic characteristics and 10% or more 10-year CVD risk.

Results

The weighted prevalence of 10% or more 10-year CVD risk was 24.70% (95% CI: 23.94%-25.47%). Participants who were currently working, living alone, and widowed had 3.63, 1.42, and 1.59 times increased odds of having a high 10-year CVD risk, respectively, after adjusting for other variables.

Conclusion

About a quarter of older adults and the elderly population in India have a 10-year risk for a fatal or non-fatal cardiovascular event of 10% or more, as estimated using a non-laboratory based chart.

## Introduction

Cardiovascular diseases (CVD), the leading cause of disease burden and deaths, require cost-effective and patient-centric approaches integrated into a country's routine primary healthcare (PHC) system [1,2]. Risk-based CVD management under the HEARTS technical package to improve cardiovascular health recommends using CVD risk charts to stratify patients for management [3]. There are two types of CVD risk charts: a) laboratory-based, which needs information on age, sex, smoking status, systolic blood pressure, status of diabetes mellitus, and total cholesterol value, and b) non-laboratory-based, which needs information on body mass index (BMI), but does not need information on diabetes mellitus and cholesterol [3]. The World Health Organization (WHO) CVD Risk Chart Working Group has recently recalibrated the charts [4]. As these charts are validated for adults aged 40-74 years, the dataset of the Longitudinal Aging Study in India (LASI), a nationally representative survey of older adults and elderly aged 45 years and above, and their spouses, would be appropriate for estimating the prevalence of CVD risk levels in India [4,5]. Hence, we aimed to estimate the 10-year risk for developing fatal or non-fatal CVD using the 2019 updated WHO CVD Risk Chart among participants of the LASI Wave-1 (2017-18) and to describe its association with socio-demographic characteristics.

## Materials and methods

This study was performed using the Wave-1 dataset of LASI, conducted during 2017-18 by the International Institute of Population Sciences (IIPS), after obtaining authorization [5]. The detailed methodology of LASI is given elsewhere [5]. As serum cholesterol was not studied in LASI, we used a non-laboratory-based CVD risk chart for South Asia to calculate CVD risk [4,6]. Individual and biomarker datasets were merged, and required variables (i.e., age, gender, self-reported current smoking status, BMI, and SBP) were selected. Of the 72,250 participants in the Wave-1 LASI, 11,621 participants were ineligible (9,904 of age < 40 or > 74 years and 1,717 with a history of CVD), and 5786 participants had at least one missing observation, giving 54,843 participants (90.45% of eligible) for analysis. CVD risk is a continuous variable and ranges from 1% to 40% [3]. The risk level was categorized as <5%, 5% to <10%, 10% to <20%, 20% to <30%, and > 30% [4,6]. Analysis was performed in Stata software (version 14.1; StataCorp LLC, College Station, Texas) after applying sample weights to all observations to account for unequal selection probabilities and non-response [7]. Weighted prevalence with 95% confidence intervals (CI) was estimated for the key outcome (i.e., the prevalence of participants with different 10-year CVD risk levels). Logistic regression analysis was performed to determine the association between socio-demographic characteristics and 10-year CVD risk levels of more than or equal to 10%. As this is a secondary analysis, ethical approval is not required. Definitions of the variables and details of ethical approval for LASI are available in the survey report [5].

## Results

The mean (SD) age of participants was 56.35 (± 8.67) years; 39% were aged more than or equal to 60 years, and 57.1% were females. One-third had a BMI of more than or equal to 25 kg/m^2^, and almost one-fourth had an SBP of more than or equal to 140 mmHg. The median (IQR) risk for fatal or non-fatal cardiovascular events among India's elderly and older adults was 6% (IQR: 3, 9). The weighted prevalence of 10-year CVD risk of more than or equal to 10% was estimated to be 24.70% (95% CI: 23.94%-25.47%), as shown in Table 1.

**Table 1:** Ten-year cardiovascular disease risk levels for participants included in the analysis (N = 54,843) CVD - Cardiovascular disease

Ten-year cardiovascular disease risk levels	% (Frequency)	Weighted % (95% CI)
< 5%	49.71 (27,263)	49.40 (48.49–50.31)
5 to < 10%	26.05 (14,289)	25.90 (25.14–26.67)
10 to < 20%	22.97 (12,599)	23.60 (22.84–24.36)
20% and above	1.27 (692)	1.10 (0.98–1.25)
Less than and 10% or above CVD risk levels		
< 10%	75.77 (41,522)	75.30 (74.52–76.06)
More than or equal to 10%	24.23 (13,291)	24.70 (23.94–25.47)

With bivariable logistic regression, monthly per capita expenditure (MPCE), current working status, living arrangement, and marital status were associated with high 10-year CVD risk with p-values of < 0.2 and were taken to a multi-variable logistic regression model. Participants who were not currently working during the survey had 3.63 times higher adjusted odds (95% CI: 3.34-3.94), persons living alone had 1.42 times higher adjusted odds (95% CI: 1.14-1.76), and people who were widowed had 1.59 times higher adjusted odds (95% CI: 1.44-1.77) of having 10-year CVD risk of more than or equal to 10% compared their counterparts, adjusted for other variables in the model. All these associations were statistically significant, with a p-value of < 0.05. Table 2 summarizes the findings of logistic regression.

**Table 2:** Logistic regression analysis between socio-demographic characteristics and 10-year CVD risk levels of participants (N = 39,265 for adjusted analysis) MPCE - Monthly per capita expenditure Currently working data were available for 39,366 participants; variables included in multi-variable logistic regression: Current working status, living arrangements, and marital status

	Ten-year CVD risk		Odds ratio (95% CI)	P-value	Adjusted odds ratio (95% CI)	P-value
	< 10%	≥ 10%				
	n (%)	n (%)				
Residence						
Rural	27,253 (75.9)	8646 (24.1)	1		-	-
Urban	14,299 (75.5)	4645 (24.5)	0.99 (0.88–1.11)	0.86	-	-
MPCE quintile						
Poorest	8,132 (75.1)	2699 (24.9)	1		1	
Poorer	8,454 (76.1)	2662 (23.9)	0.92 (0.83–1.01)	0.08	0.90 (0.80–1.01)	0.09
Middle	8,309 (75.1)	2763 (24.9)	1.01 (0.91–1.13)	0.8	1.07 (0.95–1.21)	0.23
Richer	8,365 (75.7)	2687 (24.3)	1.08 (0.93–1.25)	0.3	1.06 (0.92–1.20)	0.42
Richest	8,292 (77.0)	2480 (23.0)	0.89 (0.79–1.01)	0.07	0.92 (0.81–1.06)	0.25
Currently working						
Yes	22,675 (82)	4976 (18)	1		1	
No	6,216 (53.5)	5399 (46.5)	3.90 (3.59–4.23)	< 0.00	3.63 (3.34–3.94)	< 0.00
Living arrangements						
Living with spouse, children, or others	40,567 (76.2)	12653 (23.8)	1		1	
Living alone	985 (60.7)	638 (39.3)	2.48 (2.08–2.95)	< 0.00	1.42 (1.14–1.76)	<0.00
Marital status						
Currently married or live-in relationship	34,588 (78.8)	9281 (21.2)	1		1	
Widowed	5,833 (61.0)	3725 (38.9)	2.73 (2.46–3.02)	< 0.00	1.59 (1.44–1.77)	<0.00
Divorced, separated, or deserted	626 (79.9)	157 (20.1)	1.06 (0.78–1.44)	0.7	0.75 (0.51–1.09)	0.13
Never married	504 (79.8)	128 (20.2)	0.68 (0.25–1.23)	0.2	0.73 (0.50–1.07)	0.11

## Discussion

We found that about 25% of older adults and the elderly population in India had a 10-year risk for a fatal or non-fatal cardiovascular event of more than or equal to 10%, as per the 2019 updated WHO CVD risk chart. The prevalence of 10-year CVD risk of more than or equal to 20% was lower using the non-laboratory based CVD chart, similar to previous studies that observed poor performance of non-laboratory CVD risk chart for persons with diabetes (non-lab chart vs. lab-based chart agreement was 45% for men and 25% for women) [4,8].

About three-fourths of the countries, mainly high-income or upper-middle-income countries, reported that > 50% of PHC facilities offered cardiovascular risk stratification services. None to < 25% of PHC facilities in LMICs offered this service [9]. In a limited resource setting, the non-laboratory chart can be used to prioritize high-risk individuals (more than or equal to 10% 10-year risk) for serum cholesterol testing and a laboratory-based CVD assessment [4,10]. A study conducted in North India showed that the CVD risk estimated using laboratory and non-laboratory-based CVD charts had a good agreement (kappa value=0.64) [11]. Studies on the cost-effectiveness and feasibility of implementing risk-based CVD management by community health workers could provide new evidence [12].

Estimates of 10-year CVD risk given by Peiris et al. were lower than this study, probably because of the younger age group of participants included in the National Family Health Survey (NFHS) [8]. Estimates from the National NCD Monitoring Survey (NNMS) 2017-18 were similar to this study. However, risk levels of more than or equal to 10% were found to be different from this study, which could be due to the use of laboratory-based WHO-ISH CVD risk prediction charts (2007) in NNMS [13]. This is the first available analysis for CVD risk assessment in a large, nationally representative sample with a more appropriate age group. Also, only < 10% of all eligible participants had at least one missing variable, making the results generalizable to the population of India. Our study found that there is no significant association between CVD risk levels based on place of residence and MPCE tertiles. The socially vulnerable groups, including those who are not currently working, living alone, and those who are widowed, were identified to have significantly higher CVD risk, after adjusting for MPCE quintiles. Being a cross-sectional survey, an association of sociodemographic variables with high CVD risk could not be established, which could be examined with the availability of subsequent panel survey datasets.

## Conclusions

About 25% of older adults and the elderly population in India had a 10-year risk for a fatal or non-fatal cardiovascular event of more than or equal to 10%, as per the 2019 updated WHO CVD risk chart. These findings provide evidence for the need to plan CVD services, such as increasing the usage of CVD risk charts, scaling up cholesterol testing, and continuing access to drugs such as statins and antihypertensives in primary healthcare settings in India.

## References

[1] GBD 2019 Diseases and Injuries Collaborators (2020). Global burden of 369 diseases and injuries in 204 countries and territories, 1990-2019: a systematic analysis for the Global Burden of Disease Study 2019. Lancet.

[2] World Health Organization (2016). HEARTS: Technical Package for Cardiovascular Disease Management in Primary Health Care. https://apps.who.int/iris/handle/10665/252661.

[3] World Health Organization (2020). HEARTS Technical Package for Cardiovascular Disease Management in Primary Health Care: Risk Based CVD Management. https://www.who.int/publications/i/item/9789240001367.

[4] The WHO CVD Risk Chart Working Group (2019). World Health Organization cardiovascular disease risk charts: revised models to estimate risk in 21 global regions. Lancet Glob Health.

[5] International Institute for Population Sciences (IIPS), National Programme for Health Care of Elderly (NPHCE), Ministry of Health and Family Welfare (MoHFW), Harvard T. H. Chan School of Public Health (HSPH) and University of Southern California (USC) (2020). Longitudinal Ageing Study in India (LASI) Wave 1, 2017-18, India Report. https://www.iipsindia.ac.in/sites/default/files/LASI_India_Report_2020_compressed.pdf.

[6] World Health Organization (2023). WHO updates: Cardiovascular risk charts. https://www.who.int/news/item/02-09-2019-who-updates-cardiovascular-risk-charts.

[7] (2023). What's new in Stata 18. https://www.stata.com/.

[8] Peiris D, Ghosh A, Manne-Goehler J (2021). Cardiovascular disease risk profile and management practices in 45 low-income and middle-income countries: a cross-sectional study of nationally representative individual-level survey data. PLoS Med.

[9] World Health Organization (2020). Assessing National Capacity for the Prevention and Control of Noncommunicable Diseases: Report of the 2019 Global Survey. https://www.who.int/publications/i/item/9789240002319.

[10] Gaziano TA, Abrahams-Gessel S, Alam S (2016). Comparison of nonblood-based and blood-based total CV risk scores in global populations. Glob Heart.

[11] Ananda Selva Das P, Dubey M, Kaur R, Salve HR, Varghese C, Nongkynrih B (2022). Who non-lab-based CVD risk assessment: a reliable measure in a North Indian population. Glob Heart.

[12] Ministry of Health and Family Welfare (2011). National Program for Health Care of the Elderly (NPHCE): Operational Guidelines 2011. https://main.mohfw.gov.in/sites/default/files/8324324521Operational_Guidelines_NPHCE_final.pdf.

[13] Indian Council of Medical Research (ICMR) - National Centre for Disease Informatics and Research (NCDIR) (2021). National Noncommunicable Disease Monitoring Survey (NNMS) 2017-18. https://www.ncdirindia.org/nnms/.

